# SUPPLEMENTAL PAYMENTS AND NURSING HOME STAFFING: A CASE STUDY OF INDIANA

**DOI:** 10.1093/geroni/igac059.2155

**Published:** 2022-12-20

**Authors:** Hari Sharma, Lili Xu

**Affiliations:** University of Iowa, Iowa City, Iowa, United States; University of Iowa, Iowa City, Iowa, United States

## Abstract

Adequate reimbursements are key to improving quality in nursing homes (NHs), but Medicaid payments remain low. Indiana implemented Medicaid supplemental payment program to non-state government (NSGOs) owned NHs to boost Medicaid payment rates. Under the program, NSGOs contribute the state-share of the supplemental payments through intergovernmental transfers allowing the state to claim federal matching funds. The supplemental payments are distributed between NSGOs and NHs but little is known about whether NHs invested the supplemental payments to improve staffing levels. In this study, we examine whether NHs receiving supplemental payments, proxied by NSGO-ownership, have higher staffing hours per resident day (HPRD) for registered nurse (RN), licensed practical nurse (LPN), and certified nurse aide (CNA) compared with NHs without supplemental payments. We use facility-year data on NSGO ownership (State of Indiana), staffing levels (Nursing Home Compare), and facility and resident characteristics (LTCfocus.org) on 500 NHs from 2009 to 2017. We use difference-in-difference (DD) regressions with year and facility fixed effects to estimate the potentially causal effect of NSGO ownership on NH staffing controlling for facility and resident characteristics. DD estimates suggest that NSGO-ownership is associated with decreased staffing level across all three types of staffing. More specifically, RN staffing decreased by 0.019 HPRD (p< 0.05), LPN staffing decreased by 0.041 HPRD (p< 0,05), and CNA staffing decreased by 0.089 HPRD (p< 0.001). Policymakers should increase the transparency of supplemental payment programs and audit the expenditures to ensure that supplemental payments are tied to quality improvement.

